# Integrative genomic analysis identifies associations of molecular alterations to APOBEC and BRCA1/2 mutational signatures in breast cancer

**DOI:** 10.1002/mgg3.810

**Published:** 2019-07-11

**Authors:** Victor Trevino

**Affiliations:** ^1^ Tecnologico de Monterrey, Escuela de Medicina y Ciencias de la Salud Monterrey Nuevo Leon México

**Keywords:** APOBEC, BRCA1, BRCA2, cancer genomics, demethylases, kinesins, mutational signatures, mutations, ubiquitins

## Abstract

**Background:**

The observed mutations in cancer are the result of ~30 mutational processes, which stamp particular mutational signatures (MS). Nevertheless, it is still not clear which genomic alterations correlate to several MS. Here, a method to analyze associations of genomic data with MS is presented and applied to The Cancer Genome Atlas breast cancer data revealing promising associations.

**Methods:**

The MS were discretized into clusters whose extremes were statistically associated with mutations, copy number, and gene expression data.

**Results:**

Known associations for apolipoprotein B editing complex (APOBEC) and for *BRCA1* and *BRCA2* support the proposal. For BRCA1/2, mutations in *ARAP3*, three focal deletions, and one amplification were detected. Around 50 mutated genes for the two APOBEC signatures were identified including three kinesins (*KIF13A*, *KIF1B*, *KIF4A*), three ubiquitins (*USP45*, *UBR4*, *UBR1*), and two demethylases (*KDM5B*, *KDM5C*) among other genes also connected to DNA damage pathways. The results suggest novel roles for other genes currently not involved in DNA repair. The altered expression program was very high for the BRCA1/2 signature, high for APOBEC signature 13 clearly associated to immune response, and low for APOBEC signature 2. The remaining signatures show scarce associations.

**Conclusion:**

Specific genetic alterations can be associated with particular MS.

## 1 INTRODUCTION

Cancer is the result of a gradual accumulation of genetic alterations (Vogelstein et al., 2013). The accumulation is driven by specific genetic processes or by environmental exposures (Helleday, Eshtad, & Nik‐Zainal, 2014). For example, in a particular patient, there could be an inherited mutation in BRCA1 but also exposure to carcinogenic molecules, and ultraviolet radiation. Each of these processes imprints a particular signature of mutations because its chemical, physical, and biological processes are different (Helleday et al., 2014). Therefore, the observed pattern of mutations provides a way to recognize the chemical or biological processes that incurred in damaged cells during the tumorigenic transformation (Alexandrov et al., 2015, 2016; Alexandrov, Nik‐Zainal, Wedge, Aparicio, et al., 2013; Helleday et al., 2014).

A specific pattern of the relative frequency of mutations imprinted into DNA is known as mutational signature (MS) (Alexandrov, Nik‐Zainal, Wedge, Aparicio, et al., 2013). A MS was conveniently designed into 96 combinations of mutations formed by the mutations of C or T surrounded by one nucleotide at 3’ and 5’ direction (Alexandrov, Nik‐Zainal, Wedge, Aparicio, et al., 2013). Current MS were identified by decomposing the observed mutations of several cancer genomes (Alexandrov, Nik‐Zainal, Wedge, Aparicio, et al., 2013; Nik‐Zainal, Alexandrov, et al., 2012; Petljak & Alexandrov, 2018) and validated by comparison with the observed signatures of specific carcinogens during controlled conditions (Helleday et al., 2014). Currently, there is a catalog of around 30 different signatures (https://cancer.sanger.ac.uk/cosmic/signatures). Yet, new signatures are being discovered (Alsøe et al., 2017; Inman et al., 2018; Pilati et al., 2017) and systematically determined in vitro (Kucab et al., 2019). However, the specific factors yielding many MS are still unknown (Helleday et al., 2014). Moreover, even for those signatures of a known factor, it is unknown whether the mutational process imposes biological constraints that shift the evolution of clonal selection generating specific mutations or alterations or whether the clonal process is affected by previous alterations.

The Cancer Genome Atlas (TCGA) and the International Cancer Genome Consortium (ICGC) have analyzed several cancer types generating large amounts of cancer genomics data (Stein, Knoppers, Campbell, Getz, & Korbel, 2015). Furthermore, there are estimations of the MS available for most of the TCGA and ICGC data (Huang et al., 2018). Nevertheless, to our knowledge, there are no systematic analyses associating MS to other genomic data. This is important because it may guide novel treatments and innovative experiments for patients showing particular signatures or provide insights into possible causes, cofactors, or molecular biases for some signatures.

Here, an approach to determine associations of MS with genomics data including mutations, copy number alterations (CNA), gene expression, miRNA expression, and reverse phase protein array (RPPA) data is presented. Because a tumor is generally affected by several mutational processes (MP), the overall contribution of a particular signature is small (Alexandrov, Nik‐Zainal, Wedge, Campbell, & Stratton, 2013; Huang et al., 2018) and its approximation is subject to errors (Alexandrov, Nik‐Zainal, Wedge, Campbell, et al., 2013), the method considers only extreme values of the MS contribution. The approach is applied to more than 900 breast cancers from TCGA showing promising associations. Breast cancer was chosen because it is known to contain one of the most versatile occurrences of MS to date (Alexandrov, Nik‐Zainal, Wedge, Aparicio, et al., 2013).

## 2 RESULTS

### 2.1 Statistical estimation of association

**Figure 1:**
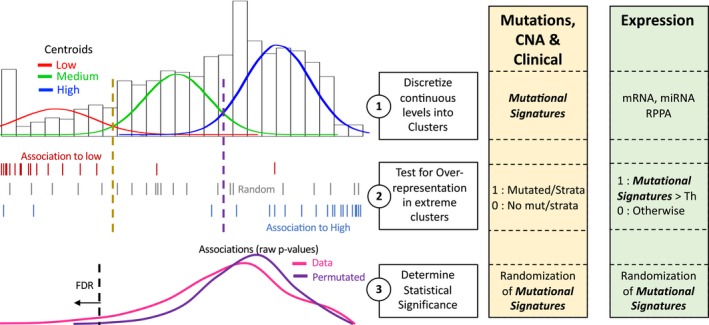
Proposed approach to associate genomics data to mutational signatures

The central hypothesis is that the extreme values of the signature could reveal specific patterns of mutations, alterations, or peaks of expression (Figure 1). Thus, for the association, an overrepresentation strategy was used in which the MS values were clustered into zero (low), medium, and high values using a k‐means algorithm and the mutations or copy alterations were counted per signature cluster. Then, the extreme clusters (zero and high) were used to estimate overrepresentation using a hypergeometric test. For gene expression association, expression values were clustered as above and the molecular signature values were set to 1 if it were higher than a threshold. Four thresholds were used (0.05, 0.1, 0.2, and 0.5; see methods for details). Then the same procedure of counting per cluster of gene expression followed by statistical testing was used. To estimate association, a permutation‐based approach was used (Figure S1), which was highly different for mutations and CNA compared to gene expression, presumably due to the continuous nature of gene expression data. Here, a cut‐off of *p* = 0.0003 equivalent to an FDR of 13% was used for mutations and CNA data, to avoid losing possible subtle associations. A cut‐off of *p* = 10^−5 ^equivalent to an FDR = 0.14% was used for expression data to avoid overwhelming associations (Figure S1). Simulations provided in supplementary material show that the described procedure may have a sensibility between 12% and 52% depending on specific scenarios suggesting that the procedure is able to capture true positives.

### 2.2 Overall results in breast cancer data

**Figure 2:**
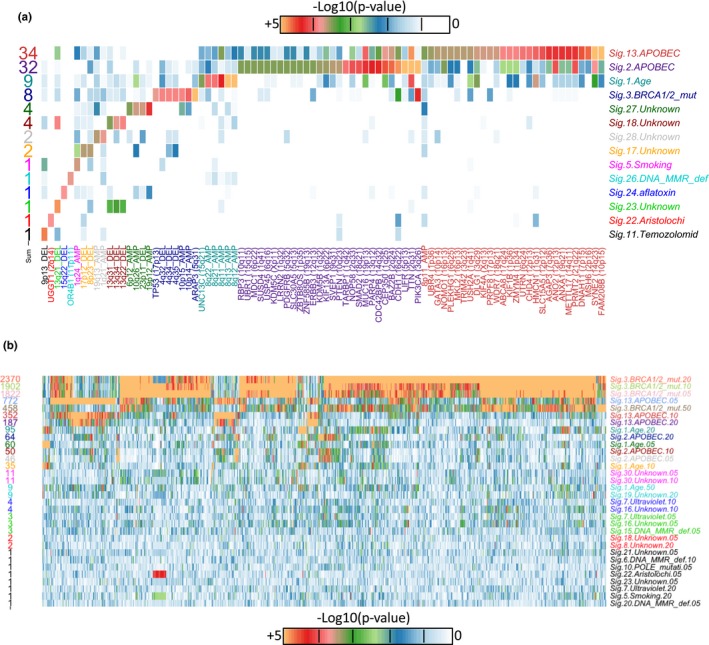
Summary of significant results. The figure shows heatmaps of *p*‐values for mutational signatures in vertical axis and mutation and copy number data in horizontal axis for (a) or gene expression data for (b). The number at the left of each heatmap shows the number of significant associations, at *p* < 0.0003 for (a) and *p* < 10^−5^ for (b)

Because it has been shown that hypermutated samples can influence the identification of significant mutations (Treviño, Martínez‐Ledesma, & Tamez‐Peña, 2017), the samples showing more than 500 mutated genes were removed before the analysis (equivalent to samples carrying more than 10 mutations per Mb approximately). From the 843 samples used, the high values of MS data do not seem correlated (Figure S2). Nevertheless, some significant correlations were observed (|r|> 0.2 and *p* < 10^−6^) involving both apolipoprotein B editing complex (APOBEC) signatures, BRCA1/2 signature, age signature, and mutation burden as shown in Figure S3. All pairs were significant except for BRCA1/2 and APOBEC signature 13. Mutation burden correlated positively to BRCA1/2 and APOBEC signature 2 and 13. On the contrary, mutation burden correlated negatively to the age signature 1. For the association with clinical and demographic data, only the HER2 immunohistochemical indicator was significantly associated with APOBEC signature 2 (Table 1). For the associations to molecular data, the overall results are summarized in Figure 2. From mutations and CNA, both APOBEC signatures show more than 30 associations, mainly to mutations. Then, age and BRCA1/2 has nine and eight associations, respectively. The detection is followed by four unknown signatures showing four or two associations. Overall, these results were specific to a MS with occasional cooccurrence mainly between APOBEC signatures. Similarly, associations to gene expressions are dominated by the BRCA1/2 signature followed by the two APOBEC signatures and then age.

**Table 1:** Maximum association of clinical and demographic data with mutational signatures^a^

Data	Top *p*‐value	Top signature
Her2 IHC = 2	0.00010	Sig.2.APOBEC
Race = Asian	0.00342	Sig.13.APOBEC
Her2 IHC = 3	0.00774	Sig.2.APOBEC
Her2 IHC = 0	0.00781	Sig.25.Unknown
Age above Q50	0.00936	Sig.5.Smoking
Age above Q75	0.02490	Sig.5.Smoking
Menopause	0.02529	Sig.22.Aristolochi
Progesterone receptor+	0.02852	Sig.3.BRCA1/2_mut
Nodes+ > 4	0.04320	Sig.27.Unknown
Latino	0.04348	Sig.4.Smoking
Nodes+ = 0	0.05064	Sig.2.APOBEC
Margin	0.05487	Sig.13.APOBEC
Race = Black	0.06402	Sig.3.BRCA1/2_mut
Cytokeratin+	0.07170	Sig.17.Unknown
Her2 IHC = 1	0.07226	Sig.6.DNA_MMR_def
Estrogen receptor+	0.07369	Sig.2.APOBEC
Race = White	0.09672	Sig.30.Unknown
Nodes+ > 0	0.10183	Sig.8.Unknown
Nodes+ ≥ 1 ≤ 4	0.14786	Sig.19.Unknown
Not Latino	0.76039	Sig.13.APOBEC

Abbreviation: APOBEC, apolipoprotein B editing complex.

a IHC, Immunohistochemistry; Q50, Quantile 50%; Q75, Quantile 75%.

The details of each signature that generated significant results will be presented below in order relative to the number of samples showing the signature.

### 2.3 BRCA mutations signature 3

*BRCA1* and *BRCA2* are involved in DNA double‐strand break repair by homologous recombination (Thompson, 2012). It has been shown that breast cancers carrying *BRCA1* or *BRCA2* germline mutations show a characteristic MS in addition to a large number of indels flanked by microhomology (Nik‐Zainal, Van Loo, et al., 2012). Some other cancers lacking *BRCA1* or *BRCA2* mutations also show this signature, probably due to cooperating genes (Alexandrov, Nik‐Zainal, Wedge, Aparicio, et al., 2013). Here, the associations for somatic mutations in *BRCA1* (*n* = 13) and *BRCA2* (*n* = 11) with the BRCA1/2 MS were *p* = 0.007 and *p* = 0.014, respectively. Because these mutations are specific for the tumor rather than germline mutations, the associations can be interpreted as true positives. Consequently, these results support the approach used and indicate that it is able to extract useful information. Nevertheless, the cut‐off adopted here for the analysis is even more stringent than those observed for *BRCA1* and *BRCA2* suggesting that higher *p*‐values may also be positive findings. Details of all *p*‐values are included in supplementary files.

**Figure 3:**
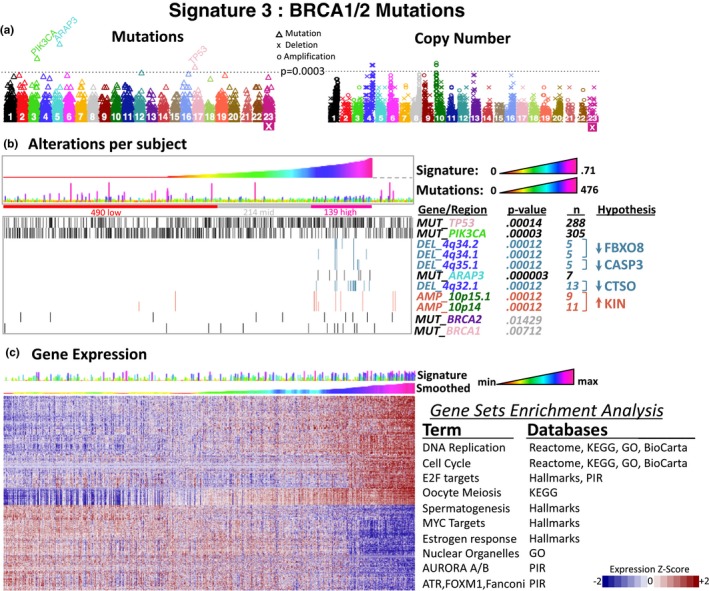
Results for the BRCA1/2 signature. (a) Manhattan plots representing the obtained *p*‐values for mutations (left) and copy number alterations (right). (b) Mutations and copy number alterations per subject sorted according to the corresponding mutational signature. (c) Gene expression sorted according to average gene expression ranks to highlight over and underexpression. The top lines represent the mutational signature values. The smoothed values were obtained by averaging a window of ±50 subjects to highlight association to mutational signatures. BRCA1 and BRCA2 in (a) were included as a reference

For genomic alterations, the results show associations in mutations for *TP53*, *PIK3CA*, and *ARAP3*, some deletions in chromosome 4, and small duplications in 10p (Figure 3).

More specifically, for mutations, there is an overrepresentation of *TP53* gene mutations for high values of BRCA1/2 signature while for *PIK3CA* there is an underrepresentation of mutations. This is consistent with previous results of mutual exclusivity between *PIK3CA* and *TP53* mutations (Kandoth, McLellan, et al., 2013). ARAP3 is a phosphatidylinositol 3,4,5‐trisphosphate‐dependent GTPase‐activating protein that modulates actin cytoskeleton and cell shape. There were seven mutations exclusively associated with high values of the BRCA1/2 MS. Recently, it has been related to cell proliferation, colony formation, migration, and invasion in papillary thyroid carcinoma (Wang et al., 2016) and to the peritoneal dissemination of scirrhous gastric carcinoma (Yagi et al., 2011). Interestingly, *ARAP3* was observed mutated in metastases of a prostate carcinoma carrying germline mutations in *BRCA1* (Nickerson et al., 2013).

**Figure 4:**
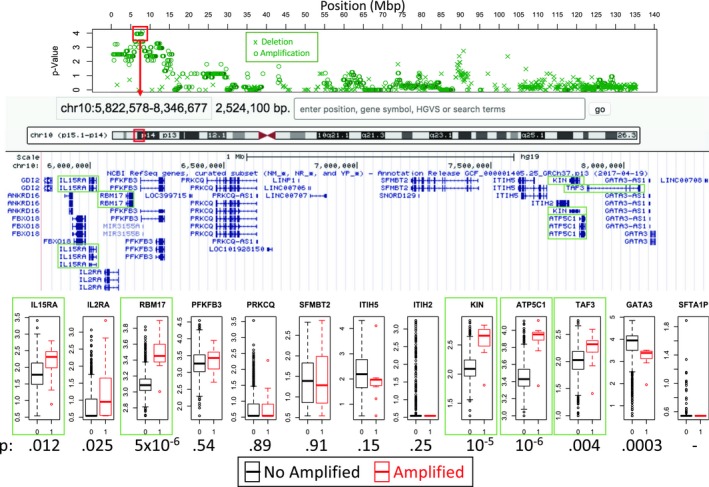
Expression of genes in the amplified region of chromosome 10 for BRCA1/2 mutational signature. The top panel shows the estimated hypergeometric *p*‐values along the chromosome. Next panel shows genes from coordinates using the Genome Browser (https://genome.ucsc.edu, GRCh37/hg19). Bottom boxplots show the expression from RNA‐Seq of nonamplified samples (in black) in comparison with amplified samples (in red). Bottom *p*‐Values were estimated using a nonparametric Wilcoxon test. Framed genes (in green) are significant

For CNA data, the duplication between 10p15.1 and 10p14 (6 Mbp–8.2 Mbp) show a peak of significance around 8 Mbp. From the genes in this region (Figure 4), *GATA3*, *KIN*, and *PFKFB3* seems interesting because of their relation to DNA damage. *GATA3* is a transcription factor frequently mutated in breast cancer (Banerji et al., 2012; Ellis et al., 2012; Network et al., 2012) and thought to be an oncogene (Smid et al., 2016). Recently, GATA3 has been implicated in homologous recombination repair by promoting CtIP (RBBP8) expression (Zhang, Tang, Jiang, & Mao, 2017), which interacts with BRCA1. KIN (*KIN17*) is a ubiquitous nuclear protein initially thought to be implicated in DNA repair (Despras et al., 2003; Masson et al., 2001). Although the mechanism is unknown, it has been seen that KIN assists double‐stranded break repair (Le et al., 2016) and that overexpression promoted DNA replication and cell proliferation (Zeng et al., 2011). *PFKFB3* encodes a 6‐phosphofructo‐2‐kinase enzyme involved in glycolysis that is able to delay cell cycle and inhibits cell growth (Calvo et al., 2006; Shi et al., 2018). Moreover, P53 improves DNA repair by suppressing *PFKFB3* expression (Franklin et al., 2016). An analysis of the expression of these genes among amplified samples shows that KIN is the best candidate of the association because of its observed overexpression compared to *PFKFB3* and *GATA3* whose gene expression does not change or even decrease in amplified samples respectively (Figure 4). Although *ATP5C1* is the most significant overexpressed gene within the region, KIN seems to be closely related to reported DNA damage mechanisms and its overexpression matches the expectation of an amplified region. *GATA3* downregulation, on the other hand, may be also implicated but indirectly caused by the amplification.

Three deleted regions were detected in chromosome 4 (Figure S4). The first deletion around 4q31.3–4q32.2 involved 27 genes also included in RNA‐seq data. From these, three genes were consistently less expressed in deleted samples, *CTSO*, *TMEM144* and *ETFDH* (Figure S4b). Literature revision supports that *CTSO* has been linked to *BRCA1* by a mechanism that modulates *BRCA1* expression in breast cancer (Cairns et al., 2017; Ingle et al., 2013). *CTSO* polymorphisms have been also linked to prognosis (Hato et al., 2016). A second deletion region around 4q34.1 including 10 genes showed concurrent lower expression of *FBXO8* (Figure S4c). Nevertheless, the role of *FBXO8* in this context is unknown. The third region around 4q35.1 involving 15 genes showed consistent expression with *IRF2*, *CASP3*, *SNX25*, *UFSP2*, and *CCDC110* (Figure S4d). Further literature analysis revealed that caspase 3 (Casp3) is highly involved in DNA damage response targeting partners of BRCA1 and BRCA2 such as RAD51 and RAD21 (Brown & Holt, 2009; Brown, Robinson‐Benion, & Holt, 2008; Martin & Ouchi, 2005).

From gene expression, the MS for BRCA1/2 was related to the highest number of genes reaching 2,805 at *p* < 10^−5 ^(Figure 3c). The clusters formed involve the two basic patterns of under‐ and over‐expression in which the last shows at least three clear subpatterns of increasing expression. A Gene Set Enrichment Analysis (GSEA) analysis shows that these genes are mainly related to terms involving DNA replication and cell cycle, which is consistent with BRCA1/2. As shown above, in the mutational analysis, *ARAP3* was associated with mutations. Nevertheless, ARAP3 has also been linked to Ras proteins (Bao et al., 2016; Krugmann, Williams, Stephens, & Hawkins, 2004). Interestingly, significant association was also observed with overexpressed Ras genes (*RHOB*, *RHBDL1*, *RHOH*, *RHOBTB2*, *RHOA*, *RHOT2*) and associated to underexpressed genes (*RHBDL2*, *RHBDF2*, *RHEB*) in relation to high values of the MS. These results further support the ARAP3 association and suggest a role through Ras pathways.

### 2.4 APOBEC signatures 2 and 13

The signatures 2 and 13 have been attributed to activation‐induced cytidine deaminase (AID) and APOBEC involved in the conversion of cytidine to uracil, which is linked to base excision repair and DNA replication (Alexandrov, Nik‐Zainal, Wedge, Aparicio, et al., 2013; Helleday et al., 2014). These signatures have been observed correlated to overall mutation burden and APOBEC3A/B expression (Alexandrov, Nik‐Zainal, Wedge, Aparicio, et al., 2013; Faden et al., 2017; Glaser et al., 2018; Wang, Jia, He, & Liu, 2018). Here, a correlation analysis across signatures revealed that the top correlation was observed between APOBEC signatures 2 and 13 (*r* = 0.55, *p* < 10^−15^, Figure S3). In both signatures, we noted a correlation to mutation burden (*r*_sig2_ = 0.32, *r*_sig13_ = 0.44, *p* < 10^−15^) in agreement with previous observations (Alexandrov, Nik‐Zainal, Wedge, Aparicio, et al., 2013; Glaser et al., 2018). A close analysis of the expression of APOBEC gene family supports strong correlations for APOBEC3 G, D, A, C, and B (*p* = 10^−11^, 10^−7^, 10^−7^, 10^−7^, 10^−5^, 10^−5^ respectively) to APOBEC signature 13 only (Figure S5). For signature 2, the associations were poor, the top associated APOBEC was APOBEC3C (*p* < 8 × 10^−4^, rank 347). These results support the analysis proposed but also highlight that gene expression of the APOBEC family of genes is mainly related to signature 13 in breast cancer.

**Figure 5:**
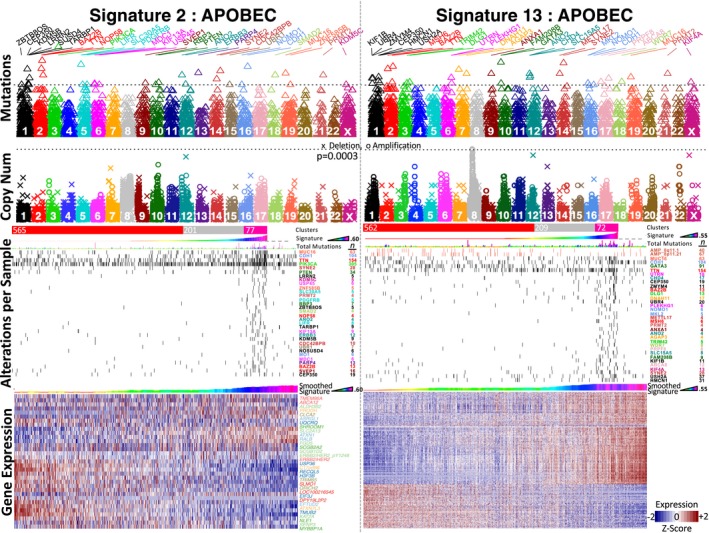
Results for APOBEC mutational signatures. Left and right panels for APOBEC signature 2 and 13. Top panels show Manhattan representations of mutations and copy number. The *p*‐value is shown in −Log10. The third panels show a summary of the significant alterations while the four panels show a selected set of associated expressed genes. APOBEC, apolipoprotein B editing complex

For CNA data, only an amplification in 8q11.1–8q11.21 was noted in the absence of signature 13. For mutations, several mutated genes were detected, mainly, in those tumor samples carrying a high number of mutations (Figure 5). Thirty‐two significantly mutated genes were observed in each signature. The set of genes include a subset that was detected in both signatures (*TTN*, *CDH1*, *BAZ2B*, *CEP350*, *MUC16*, *ANO2*, *PRMT2*, *SYNE2*, *NOMO1*). In both signatures, genes participating in DNA damage or APOBEC pathways were evident like *PIK3CA*, *CDH1*, *SMAD2*, *PTEN*, *KDM5C*, *KDM5B*, *ERBB3*, *USP45*, and *MDC1* for signature 2 and *MSH6*, *CHD4*, *GATA3*, and *CDH1* for signature 13 (Figure 5).

Because there is a correlation of the APOBEC signature to mutation burden, a systematic literature revision of genes related to hypermutation was performed. This revision emphasized *MSH6*, which as also been observed mutted in hypermutated tumors in gliomas, prostate, and colorectal cancers (Cancer & Atlas, 2012; Johnson et al., 2017; Pritchard et al., 2014).

Some mutations in *PIK3CA* have been associated with the activity of the APOBEC enzymes (Henderson, Chakravarthy, Su, Boshoff, & Fenton, 2014). In agreement with the results here, mutations in *PIK3CA* were overrepresented in high values of the APOBEC signature 2. Intriguingly, in the same pathway, *PTEN* mutations, also in signature 2, were also overrepresented (Figure 5). However, *PTEN* and *PIK3CA* mutations were neither comutated (*X^2^* test, *p* = 0.85) nor mutually exclusive (Comet test, *p* = 0.35). Yet, *PTEN* has been involved in the regulation of AID transcription in germinal center B cells in mice (Wang, Liu, et al., 2018). This observation suggests that *PTEN* has the potential to regulate AID and may influence APOBEC mechanisms in breast cancer perhaps independently of *PIK3CA*.

*MDC1*, mediator of DNA damage checkpoint 1, is a well‐known gene involved in DNA damage responses (Jungmichel & Stucki, 2010). Nevertheless, to our knowledge, MDC1 has not been linked to APOBEC mechanisms. Here four mutations were observed exclusively in APOBEC signature 2 tumors suggesting a possible implication. *ERBB3* is a member of the family of epidermal growth factors receptors that includes EGFR (*ERBB1*), *ERBB2*, *ERBB3*, and *ERBB4*. Their role in breast and lung cancer is well documented (Wang, 2017). In addition, significant *ERBB3* mutations have been found in cervical cancer where no link to APOBEC is apparent (Cancer Genome Atlas Research Network et al., 2017). Here eight of 12 mutations appeared associated with the signature. *PDGFRB*, platelet‐derived growth factor receptor beta, has some evidence linking DNA damage in gliomas (Squatrito & Holland, 2011) and fibrosarcoma models (Medová, Aebersold, & Zimmer, 2013). Interestingly, there were five mutations exclusively found in APOBEC MS 2 suggesting a possible implication in breast cancer. An enrichment analysis using EnrichR highlighted the histone demethylase activity of *KDM5B* and *KDM5C* that both demethylate H3 Lys‐4. The role of KDM5B in DNA damage is relatively well known (Xu et al., 2018). Recent evidence also suggests a role for both KDM5 proteins in immune response repression (Wu et al., 2018). This result agrees with the gene expression analysis for signature 13 that showed enrichment in many biological terms associated with the immune response. Although these demethylases have not been linked functionally, remarkably, the nine and five mutations corresponding to *KDM5B* and *KDM5C* were mutually exclusive suggesting functional cooperation and possible role in APOBEC. In addition, other members of the gene family have been linked to cancer survival such as *KDM6A* and *KDM3A* (Treviño et al., 2017). *KDM6A* was significant in pediatric brain cancer and marginally significant in adenocarcinomas of the lung and stomach while *KDM3A* was marginally significant in stomach adenocarcinoma. USP45 (ubiquitin specific peptidase 45) is linked to DNA damage by removing ubiquitylation of ERCC1, a well‐known excision repair gene, and finally promoting DNA repair (Perez‐Oliva et al., 2015). Cells lacking USP45 are highly sensitive to UV damage presumably due to mutation aggregation. The five mutations exclusively observed in APOBEC mutational breast tumors suggest a dysfunctional USP45 contributing to the accumulation of mutations.

Two ubiquitins that are part of the N‐end rule pathway (Varshavsky, 2011), *UBR1* in signature 2 and *UBR4* in signature 13 were detected (*n* = 5 and *n* = 17 mutations, respectively). UBR1 has been related to DNA repair due to its interaction with RAD6 (Hwang, Shemorry, & Varshavsky, 2009). The total mutations in both genes, seven in *UBR1* and 20 in *UBR4*, were mutually exclusive suggesting a similar role and a connection with APOBEC mechanisms.

Kinesin family of proteins is involved in transporting cargo through cells. Some kinesins have already been linked to DNA damage responses (Lottersberger, Karssemeijer, Dimitrova, & de Lange, 2015; Mekhail, 2018; Sheng, Hao, Yang, & Sun, 2018). Interestingly, three kinesins, *KIF1B* and *KIF4A* in signature 13 and *KIF13A* in signature 2, were detected associated with APOBEC signatures (Figure 4). KIF4A is localized in the nucleus playing a role in DNA damage responses related to BRCA2 (Wu et al., 2008) and other proteins (Sheng et al., 2018). Remarkably 31 of 32 tumors showed mutually exclusive mutations considering these three genes (11, 12, and 8 for *KIF1B*, *KIF4A*, and *KIF13A* respectively). Intriguingly, the expression of *KIF1B* and *KIF4A* is more significantly associated with BRCA1/2 MS (*p* = 2 × 10^−6^ and *p* = 1 × 10^−8^ respectively) than to APOBEC signatures (*p* = 0.01 and *p* = 2 × 10^−4^ respectively). The expression of *KIF13A*, in contrast, is not significantly associated with the BRCA1/2 signature (*p* = 0.39) but shows a tendency with APOBEC signature 2 (*p* = 0.0003) where it was shown mutated.

SYNE2, spectrin repeat containing nuclear envelope protein 2, also called nesprin‐2, binds to the cytoplasmic F‐actin tying nucleus to the cytoskeleton (Rashmi et al., 2012). *SYNE2* has been found frequently mutated in urothelial cancer cell lines (Nickerson et al., 2017). Recently, SYNE2 has been associated with DNA damage responses (Warren et al., 2015), possibly through BRAP2, a BRCA1 associated protein (Davies, Wagstaff, McLaughlin, Loveland, & Jans, 2013). Interestingly, SYNE2 and KIF proteins participate in the LINC complex (LInker of Nucleoskeleton and Cytoskeleton) (Stroud, 2018), which has been hypothesized to be involved in the movement of DNA breaks within the nucleus to reach repairing complexes at the nuclear pore (Mekhail, 2018). Here, more than 20 mutations in *SYNE2* were overrepresented in APOBEC signature 13.

PARP4, polymerase family member 4, add ADP‐ribose to proteins. Recently, *PARP4* has been found to be mutated in patients having thyroid and breast cancer (Das, Kundu, Laskar, Choudhury, & Ghosh, 2018). Here, at least 10 of 13 mutations were found in high values of APOBEC signature 2.

ANXA1, annexin 1, is a membrane protein that binds phospholipids. It has been associated with DNA damage responses (Park, Lim, & Baek, 2015; Swa, Blackstock, Lim, & Gunaratne, 2012). Here, four mutations were observed in the APOBEC 13 signature.

PRPF8, pre‐mRNA processing factor 8, is an important component of the spliceosome (Růžičková & Staněk, 2017). Recently, it has been related to homology‐directed DNA repair in BRCA1‐driven homologous recombination (Onyango, Lee, & Stark, 2017). Here, six of eight mutations occurred in tumors carrying APOBEC MS 13.

PRMT2 methylate arginine residues on histones and target transcription factors. The knock‐down of the *PRMT2* mRNA increase the expression of nucleotide excision repair and homologous recombination DNA repair genes (Oh et al., 2014). So, PRMT2 may participate in DNA repair connected to APOBEC activity. Here, four mutations were observed in signature 2.

*LIFR* encodes the receptor of LIF. LIF overexpression inhibited DNA damage responses besides other functions (Liu et al., 2013). Inoculation of LIFR that sequester LIF reversed the effects (Liu et al., 2013). Here, eight mutations were observed in APOBEC signature 2, which suggests that some mutations may overactivate LIFR increasing the mutation rate.

Analysis of associations of mutated genes to biological terms highlighted that six mutated genes (*GATA3*, *SMAD2*, *CEP350*, *KIF1B*, *USH2A*, *MSH6A*) can be significantly regulated by BRCA1 (EnrichR analysis over PPI transcription factors, *p* = 0.0001415, adjusted‐*p* = 0.01). This result together with the analysis of other mutated genes above suggests a connection between APOBEC signature and BRCA1 responses.

Because APOBEC signatures were correlated to mutation burden, it can be expected that mutated genes may be also comutated just by chance. Nevertheless, there are examples where comutation is functional. For example, deletions in *CDH1* and mutations in *PIK3CA* induce an immune subtype of breast cancer in a mice model (An et al., 2018). Therefore, a systematic revision of comutation and mutually exclusive mutations were performed. The results show significance for comutation but not for mutual exclusivity. The interesting clusters of comutated genes include not only a significant comutation between *CDH1* and *PIK3CA* as above but also between *ZMYM4* and *UBR4* or between *MSH6* and *MKL2* (Figure S6). Comutations between more than two genes from a set of 32 genes seem very unlikely (*p* < 0.03) and therefore can be presumably attributed to mutation burden. This may be the case for three clusters in APOBEC signature 2 (BAZ2B/LIFR/NOP58, UBR1/SUSD4/NOMO1, and TTN/SYNE2/USP45) and four clusters in signature 13 (BAZ2B/DLG1/DNAH11/NOMO1, WDR7/METTL17/MUC16, and TTN/USH2A/HMCN1).

To estimate the fraction of subjects carrying mutations in these signatures, only the genes that show a profile highly exclusive for the MS were considered (28 genes that show less than 40 mutations). There were 22% or 33% of subjects having at least one mutation in any gene from signature 2, or 13, respectively, suggesting that a considerable proportion of the activity may be explained by these genes.

Note that the hypermutated samples were removed for the analysis. Because APOBEC signatures are also highly correlated to mutation burden, it was tested whether the detected mutated genes are dependent on remaining highly mutated samples. Therefore, a rerun was performed removing tumors having more than 90 mutated genes. For signature 2, only the highly mutated *PIK3CA* remained overrepresented in high values of the signature. However, for the signature 13, an amplification in 17q12 (37.3–38.0 Mbp) was detected that includes *ERBB2*, and a positive association with four mutations in MTSS1, which has been proposed as a metastasis driver gene in melanoma (Mertz et al., 2014).

For gene expression, there were 33 and 466 genes, miRNAs, or RPPA data associated to the APOBEC signature 2 or 13, respectively (at *p* < 10^−6^ as in Figure 4; or 113/873 at *p* < 10^−5^). Although four were detected associated in both signatures (*CLCA2*, *PRODH*, *TMEM86A*, RPPA_ ERBB2|HER2_pY1248), most associations were signature‐specific indicating that the underlying processes are quite different even though the signatures are correlated. Similar results were observed in the significant mutations described above. For signature 13, high values of ERBB2|HER2_pY1248, and CASP7_cleavedD198 from RPPA data were associated to high values of the signature while *ESR1* (ER‐alpha and ER‐alpha_pS118) were associated to low values of the APOBEC signature. A GSEA analysis showed 864 gene sets significant (at FDR < 5%) for signature 13 (Supplementary Files). The terms are highly associated with immune response along with several databases followed by cancer gene sets or signatures, and cell cycle terms. For signature 2, the terms were related to region 17q25, collagen fibril organization, and four cancer signatures only.

### 2.5 Age signature 1

Signature 1 has been associated with age in almost all cancer types (Alexandrov et al., 2015). In breast cancer, this association was shown to be highly significant (Alexandrov et al., 2015).

Using the proposed approach, a strong amplification signal at 8q was noticed in CNA consisting of three regions (Figure S7a). Further analysis revealed three significant regions, a large region (20 Mbp) in the long arm of chromosome 8 close to centromere spanning 8q11.1 (47.5 Mbp) to 8q13.3 (71.7 Mbp), a small region between 8q21.11 (77.5 Mbp) and 8q21.12 (80.1 Mbp) and a third region between 8q22.1 (93.4 Mbp) and 8q22.2 (99.2 Mbp). The patients involved in these regions were 39–60 (representing 4.2%–6.4%), 61 (6.5%), and 65–82 (6.9%–8.7%), respectively. The genes within these regions that were also included in RNA‐Seq data corresponded to 71, 4, and 27 genes, of which, 38, 1, and 10, respectively, were consistently more expressed in amplified samples (raw *p* < 0.05, Wilcoxon test) supporting a possible implication (Figure S7). In contrast, the TCGA Firebrowse reports 15 focal deletions and three focal amplifications associated with age at diagnosis (years to birth) for breast cancer (https://doi.org/10.7908/C1D799SN). Within these, the closest amplification reached a *p*‐value of 0.026 at 8q24.21 (~130.9 Mbp) in our analysis, which indicates that the age signature and age, although correlated, are not interchangeable.

Besides *TTN*, mutations in *UNC13C*, *DLG1*, and *HMCN1* were associated with low or absent values of the age signature. From gene expression, there were 47 genes associated at *p* < 10^−6^ (Figure S7d). From these, 34 display positive correlation while only 13 show a negative correlation.

### 2.6 Other signatures

#### 2.6.1 DNA mismatch mutation repair

Multiple signatures have been related to defects in DNA mismatch repair (Alexandrov, Nik‐Zainal, Wedge, Aparicio, et al., 2013) (signatures 6, 15, 20, and 26). From these, signature 6 is commonly present in many cancer types (Alexandrov, Nik‐Zainal, Wedge, Aparicio, et al., 2013). In this analysis, the associations were scarce to all DNA mismatch mutation repair signatures. There were no associations to CNA data. Only three expressed genes were associated with signature 15, and one expressed gene to signatures 6 and 20. Nevertheless, for mutations, *MYO5B* and *ZCCHC12* were associated with signature 20 (*n* = 12 and *n* = 5 mutations respectively). Interestingly, another gene of the myosin family, MYO3A, was close to significance (*p *= 0.001, *n* = 8 mutations).

#### 2.6.2 POLE

Signature 10 has been related to defects in polymerase epsilon (Cancer & Atlas, 2012; Kandoth, Schultz, et al., 2013). No mutations nor CNA were associated at the *p*‐value threshold used. The topmost associated expressed gene, which was marginally significant, was *NT5M*. This is a mitochondrial enzyme that dephosphorylates the 5′‐ and 2′(3′)‐phosphates of uracil and thymine that is thought to have an effect in mitochondrial DNA replication (Rinaldo‐Matthis, Rampazzo, Reichard, Bianchi, & Nordlund, 2002). Lower expression of NT5M is associated with higher values of POLE signature. From gene expression data, none of the POLE family of genes (*POLE*, *POLE2*, *POLE3*, and *POLE4*) was associated. Marginally significant mutations were observed in *TAF1L*, *GRHL3*, *EGR3*, *DLL1*, and a deletion in 5p13.2.

#### 2.6.3 Aflatoxin

Signature 24 has been related to adducts caused by benzo‐a‐pyrenes and aromatic amines such as aflatoxin (Helleday et al., 2014). One deletion in five individuals in 15q22.2 (~59 Mbp) was observed that includes MYO1E whose gene expression is marginally downregulated (*p* = 0.09, Wilcoxon test).

#### 2.6.4 Ultraviolet

Signature 7 has been highly associated with melanoma and ultraviolet (UV) exposure (Alexandrov, Nik‐Zainal, Wedge, Aparicio, et al., 2013; Helleday et al., 2014). Although 461 tumors had an estimated component of the ultraviolet signature, scarce associations were observed, mainly two expressed genes (ZBTB7C and TTC12). Nevertheless, the sixth in rank (at *p* = 10^−5^) was ERBB2 from RPPA data (ERBB2|HER2_pY1248), showing a positive correlation. Interestingly, there is evidence linking ultraviolet radiation to increased ERBB2 activity (Han, Lim, Choi, & Kang, 2008; Madson, Lynch, Tinkum, Putta, & Hansen, 2006). The induction is thought to be related to reactive oxygen species, hydrogen peroxide, and others (Martínez‐Carpio & Trelles, 2010) in which some lipids, like squalene and cholesterol, have been implicated (Kostyuk et al., 2012). The top association for mutations was slightly below significance for *NEURL4*, which is a modulator of centrosome architecture (Al‐Hakim, Bashkurov, Gingras, Durocher, & Pelletier, 2012). There are four mutations observed in patients with very high values of the ultraviolet signature. Recently, NEURL4 has been implicated in the regulation of the TP53 activity (Cubillos‐Rojas, Schneider, Bartrons, Ventura, & Rosa, 2017).

#### 2.6.5 Alkylating agents

The alkylating agent signature 11 has been highly related to the use of temozolomide in glioblastoma and melanoma (Alexandrov, Nik‐Zainal, Wedge, Aparicio, et al., 2013; Olivier et al., 2015). In breast cancer, the common alkylating agents are cyclophosphamide and cytoxan (Zhao, Yang, Haslam, & Schwartz, 2014). Nevertheless, the genomic data were obtained before treatment. Thus, as expected, in the data used, no association was observed between the alkylating agent MS higher than zero and the use of the above drugs (*X^2^* test, *p* = 0.2776) suggesting that the estimated signature can be the result of additional drugs or biological processes. The top associations found in this analysis were related to three deletions on 9p13.2. Although three deletions seem scarce, they are sufficient to show statistical significance given that few samples carry this signature (≤121 samples, Figure S2). The region includes around 15 genes (from *RNF38* to *ALDH1B1*), of which, *SHB*, *ZCCHC7*, *POLR1E*, and *TOMM5* were expressed at a lower levels (*p* < 0.05, Wilcoxon test). Intriguingly, another gene within this region, *MELK*, seems to be related to DNA repair or temozolomide responses. *MELK* has been seen as highly expressed in cells after treatment with temozolomide (Joshi et al., 2013). In addition, MELK overexpression has been associated with poor outcome in some cancers (Huang et al., 2017; Kohler et al., 2017). Nevertheless, in basal breast cancers, it was shown that MELK is not needed for proliferation (Huang et al., 2017). The median expression of MELK was apparently lower in the three patients showing the deletion but it was not statistically significant (*p* > 0.05) suggesting that the region may be involved in breast cancer but the gene identity is uncertain.

#### 2.6.6 Smoking

Signatures 4 and 5 have been highly related to smoking and lung cancer (Alexandrov, Nik‐Zainal, Wedge, Aparicio, et al., 2013; Olivier et al., 2015). There was an amplification associated with signature 5 in 1q24.1. POGK and TADA1 were highly expressed in amplified samples.

#### 2.6.7 Unknown signatures

For many signatures, it is still unknown which is the causal agent (signatures 17, 18, 23, 27, and 28). Few associations were found, which are listed in Table 2.

**Table 2:** Summary of significant findings in breast cancer

Signature	*n*	Mutations	Amplifications	Deletions	Expression^b^
1. Age	728	HMCN1, UNC13, DLG1	8q11–8q13, 8q21.1, 8q22.1−8q22.2		153/47
2. APOBEC	451	[32 genes]			113/33
3. BRCA 1/2 mutation	468	ARAP3	10p14, 10p15.1	4q32, 4q34, 4q35	2,805/1925
4. Smoking	176				
5. Smoking	121		1q24.1		1/0
6. DNA MMR					1/0
7. Ultraviolet	461	NEURL4^a^			8/2
8. Unknown	81				2/0
9. Immunoglobulin Hypermutation	52				
10. POLE mutation	242				1/0
11. Temozolomide	121			9p13.2	
12. Unknown	96				
13. APOBEC	377	[32 genes] + MTSS1	8q11.1–8q11.21, 17q12		873/467
14. Unknown	65				
15. DNA MMR	272				3/0
16. Unknown	91				7/0
17. Unknown	138		6q12	17p12, 8p23.1, 8p21.3	
18. Unknown	203			13q21.2–13q22.2, 13q34	2/1
19. Unknown	104				9/1
20. DNA MMR	122	MYO5B, ZCCHC12			1/0
21. Unknown	174				1/1
22. Aristolochic Acid	206	UGGT1			1/0
23. Unknown	88			13q21.32–13q21.33, 13q33.2–13q34	1/0
24. Aflatoxin	278			15q22.2	
25. Unknown	70				
26. DNA MMR	86				
27. Unknown	71		19p12, 6p12.3, 10q26.3	23p11.21	
28. Unknown	103		1q21.3, 19q13.1		
29. Tobacco Chewing	177				
30. Unknown	122				18/4

Note

*n* denotes the number of samples having a value larger than 0.

Abbreviation: APOBEC, apolipoprotein B editing complex.

a Top alteration marginally significant.

b Alterations at two *p*‐thresholds (*p* = 10^−5^/*p* = 10^−6^).

## 3 DISCUSSION

Specific cancer treatments are being used and predicted to be used for specific mutations, overexpression, or subtypes (Rubio‐perez et al., 2015). The association of molecular data to MS can provide important insights regarding possible causes, cofactors, or novel treatments (Cho et al., 2018; Glaser et al., 2018; Inman et al., 2018; Viel et al., 2017). Indeed, the MS by its own may be the target of specific therapies (Nickoloff, Jones, Lee, Williamson, & Hromas, 2017). Therefore, methods and analysis exploring possible associations between MS and molecular data are valuable. Nevertheless, the estimated contribution of most molecular signatures to particular tumors is generally small (Figure S2) and subject to errors (Alexandrov, Nik‐Zainal, Wedge, Campbell, et al., 2013). Consequently, here we used extreme values of the MS to test associations avoiding those samples that could be influenced by small errors in the estimation of the MS (Figure 1). The detected known association such as mutations in *BRCA1* and *BRCA2* and the expression of APOBEC gene family within their respective signatures support the approach used. Moreover, the analysis and the review of literature provided, though speculative, show clear examples of supporting evidence that some genes are likely to be the result of positive associations.

In general, the detection of associations was low, mainly dominated by gene expression, then CNA, RPPA expression, and finally mutations (Table 2 and Figure 2). A correlation was observed between the number of samples carrying a MS and the associations found. For example, the top four most frequent MS (Age, BRCA1/2, APOBEC 2 and 13) had the highest number of associations to genomic features (Figure 2 and Figure S2). Within these, the signature for age had the lowest associations despite being present in most tumors. One reason for this result is that these tumors are more heterogeneous than those dominated by a specific signature. This is also supported by MS data (Figure S2) where tumors carrying a major component of the Age signature seem also to carry considerable components of other signatures (for example BRCA1/2, APOBEC, and DNA mismatch repair signature 6).

APOBEC, BRCA and Age signatures show an important number of associations. For mutations, only the APOBEC signatures show high numbers of associations. The correlation to the high number of mutations observed in these tumors raises the question whether the detections are the consequence of the mutation burden (false positives), the result of evolutionary pressures imposed by a broken APOBEC pathway (true positives), or the contribution of these mutations for raising the APOBEC signature (true positives). The literature revision of the genes involved provides confidence that some of the results are potentially true positives. Diverse pieces of evidence suggest similitudes in molecular mechanisms but also differences across APOBEC signatures. Three results support similitudes. First, it was observed that the contribution of APOBEC signatures across patients is correlated. Second, mutations in nine genes appear associated with both signatures, perhaps due to the inherent correlation. Finally, some gene families seem to be detected in both signatures such as histone demethylases (KDM5B/C), ubiquitins (*UBR1/4*), and kinesins (*KIF1A/4A/13A*). Nevertheless, clear differences between associations were also observed. For example, 46 out of the 55 mutated genes are specific for their corresponding signature. Furthermore, APOBEC signature 13 shows expression correlation to far more genes (including APOBEC) than signature 2. Thus, overall, similitudes and differences may provide future directions of research to elucidate precise mechanisms between these signatures.

Only somatic mutations were analyzed and this has some limitations. For example, in signature BRCA1/2, there were supporting associations to somatic mutations in *BRCA1* and *BRCA2*. A closer look of the raw files suggests that these somatic mutations were present in a single allele, which is contradictory to the fact that a single functional copy in these genes is sufficient for normal homologous recombination (Scully & Livingston, 2000). Nevertheless, an analysis of the same TCGA data has revealed that most of the carriers of *BRCA1* and *BRCA2* somatic mutations show loss of heterozygosity (LOH) either because of deletions or epigenetic silencing (Polak et al., 2017), which can be considered. This suggests that systematic analyses using more complex estimations are needed to separate mutations under LOH, appearing as biallelic, and those monoallelic.

The major component of around 20% of the breast tumors was not Age, APOBEC, or BRCA1/2 signatures but distributed across other signatures. The range goes from 44 samples whose major component is signature 6 (DNA mismatch repair) to 14 samples for smoking, 11 samples for ultraviolet and tobacco chewing, and only two samples for *POLE*, among others. It would be interesting to compare with different populations or subtypes of breast cancer whether there are differences in the distribution of MS and resulted associations. This may encourage researchers to study specific subtypes of breast cancer tumors enriched in particular MS. For example, breast cancer tumors from populations where sunlight exposure is more frequent, perhaps those closer to the equator, may show higher components of the ultraviolet signature and may be suitable to study associations in this particular signature.

Except for those hypermutated, all samples were used. On the contrary, low‐mutated samples may show higher errors in the estimations of the MS weights. Nevertheless, low‐mutated samples will barely contribute mutations and therefore have low effects for false positive calls. Low‐mutated samples may, however, subtly contribute to false negatives inflating the number of samples in clusters. In this context, only 5% of samples showed less than 10 mutated genes, thus, the negative effect of low‐mutated samples seems to be low. Future analyses may need to explore and compare the results with and without removing the low‐mutated samples.

Many mutated genes, CNA, and gene biased expression were identified. Thus, the approach and results delivered could serve to prioritize future investigations on the contribution of specific genes or alterations.

The approach provided has been applied here to breast cancer, nevertheless, in principle, it can be applied to any other cancer or dataset. Therefore, it can be useful to discover and test novel associations in other cancers and to identify generic features in many cancer types.

## 4 CONCLUSION

The estimated MS are proxies of the evolutionary pressures and exposures encountered by tumors during progression. The identification of molecular alterations associated with MS may help to study and reveal the biological mechanisms involved. Therefore, methods that detect possible associations between MS and molecular data are valuable. Nevertheless, the estimation of the contribution of MS to a particular patient is low and affected by methodological errors. Here we used a three‐centroid method that focuses on extreme values of the MS for testing associations avoiding those samples that could be influenced by small errors in the estimation of the contribution to a MS. The detected known association such as mutations in *BRCA1* and *BRCA2* and the expression of APOBEC gene family within their respective signatures support the approach used. The analysis of the literature shows examples of evidence that support plausible associations in breast cancer. The approach provided can be used or adapted to analyze other cancer types or experiments regarding MP.

## 5 DATA AND METHODS

### 5.1 Algorithm

A scheme of the analysis is shown in Figure 1. The approach is based on observing many mutations in patients showing a high‐valued MS and few or none mutations in patients not showing the MS (zero‐valued). For this, three clusters were generated by the k‐means method initialized with the minimum, mean, and maximum observed MS values. Then the first and last clusters were used to estimate overrepresentation of mutations or alterations using a hypergeometric test. Only samples showing a nonmissing estimation of the MS were used even when its value was zero. Finally, a permutation‐based procedure was used to estimate statistical significance.

#### 5.1.1 Analysis of mutations and CAN

The MS was clustered by k‐means as described above while CNA and mutations were counted per signature cluster. Amplification and deletions were analyzed separately.

#### 5.1.2 Analysis of gene expression (mRNA, miRNA, RPPA)

The gene expression values were grouped into three clusters by k‐means as described above. Then MS were converted to binary values and counted across clusters for statistical test. To binarize MS, each value was set to 1 if it was higher than a threshold and 0 otherwise. To avoid threshold dependency, four thresholds were used, 0.05, 0.1, 0.2, and 0.5.

#### 5.1.3 Analysis of clinical data

Numerical and nonnumerical indicators were stratified to 1 or 0 depending on values creating dummy variables, which is similarly done in linear models. Age, estrogen receptor, progesterone receptor, cytokeratin, Her2, nodules, margin, menopause, and race were used. Age was thresholded in two quantiles (50% and 75%) setting 1 to those higher than 50% or those higher than 75%. Her2 values of 3, 2, 1, and 0 were used specifically setting 1 for those valued to 3, and so on. Nodules were stratified to larger than 4, between 1 and 4 and none. Race was stratified for white, black, Asian, and Latino.

### 5.2 Statistical estimation

To estimate a cut‐off and determine significance, MS and corresponding data were randomized before discretization. Ten permutations were performed. FDR was estimated by dividing the average number of raw *p*‐values obtained from the permutated experiment by the maximum of itself and the observed raw *p*‐values from data. Figure S1 shows the estimation of the hypergeometric *p*‐values obtained from the data and for the permutations. It also shows and supports the FDR estimations. For mutations, an additional filter was used to remove genes mutated in less than three samples.

### 5.3 Breast cancer data

The TCGA breast cancer data were downloaded from FireBrowse (http://firebrowse.org) and TCGA data portal (https://portal.gdc.cancer.gov/) around January 2017. The data included somatic mutations (MAF), somatic copy number estimations (SNP6), tumor mRNA sequencing (mRNASeq level 3), microRNA sequencing (miRSeq level 3), RPPA, and clinical information. For mutations, only genes having more than two somatic mutations were considered before further filters. Only primary tumor samples having data for mutations, CNA, and mRNA expression were used (miRSeq and RPPA data were optional). Quantile normalization was performed in mRNASeq, miRSeq, and RPPA. The MS estimations were obtained from mSignatureDB (Huang et al., 2018). mSignatureDB used the deconstructSigs (Rosenthal, McGranahan, Herrero, Taylor, & Swanton, 2016) package, which finds the weight of each of the 30 MS operating in a tumor sample. The weights obtained by deconstructSigs are highly correlated to weights deconvoluted from de novo analyses and therefore are, overall, highly reliable (Rosenthal et al., 2016). In each of the analysis, only samples showing an estimation of the MS from mSignatureDB were used. To account for tumor clonal heterogeneity and purity less than 100%, a cut‐off of ±0.5 was used to estimate amplification or deletion from CNA data. CNA data were also reduced if neighbor coordinates contained equivalent information. That is, data were merged if the differences were only one sample. An “OR” operator was used for merging. Only CNA data having more than two alterations were considered. Overall, mutation, amplification, and deletion data included 8,610 genes, 9,994 regions, and 5,262 regions respectively accounting for 23,920 binary alterations. For expression, 226 proteins were used from RPPA, 125 miRNAs, and 20,531 genes for mRNA. In total, 938 samples were included, of which 843 also contained an assigned value of MS in mSignatureDB. Because it is known that hypermutated samples may generate false results (Treviño et al., 2017; Treviño & Tamez‐Pena, 2017), the analyses were carried out filtering hypermutated samples removing those having more than 500 genes mutated.

### 5.4 Other functional and statistical analysis

EnrichR or GSEA were used to summarize the associations of gene expression (Kuleshov et al., 2016; Subramanian, Kuehn, Gould, Tamayo, & Mesirov, 2007; Subramanian et al., 2005). EnrichR test for statistical overrepresentation of a gene list within collections of genes including pathways, gene ontologies, and transcription factors (Kuleshov et al., 2016). GSEA tests the ranks of a list of genes comparing them to the rank of an experiment or collection of genes (Subramanian et al., 2007, 2005). It estimates an enrichment score which is interpreted as a significant association of ranks. The estimated *p*‐values transformed to negative logarithm were used as the indicator of the rank. The results were manually reduced considering mainly pathways, networks, gene ontology terms, and cancer hallmarks. Mutual exclusivity of mutations was tested using CoMEt (Leiserson, Wu, Vandin, & Raphael, 2015). To estimate gene comutation, a chi‐square test was used. To determine the significance of comutations, mutations were randomized per gene across the entire dataset to build a null‐distribution from the aggregate of 100 dataset randomizations. Briefly, based on the estimated null‐distribution, the significance was determined if the raw *p*‐value was less than 0.001. As an approximation, mutation burden was estimated as the number of nonsilent mutations in coding genes for consistency with all analyses that used nonsilent mutations only.

### 5.5 Validation in simulations

To support findings, some simulations were performed using simple models. The central idea was assessing the behavior of the proposed method in varied scenarios. Specifically, MS were simulated by an additive linear model formed by four components using random variables. These components represent mutations, CNA, and gene expression. Then, the proposed method was executed and the performance was assessed by sensibility, counting the number of variables detected as significant that were also used to generate the MS. A number *G* of mutated genes, the same *G* number of regions for CNA, and *G* overexpressed plus *G* underexpressed genes were used in each simulation. Thus, the model contained 4G variables. In brief, if a subject shows alteration in any chosen gene or region, it receives a positive value of the MS. For mutations and CNA, this is straight forward. For gene expression, if a subject were within the top *T*% of the most expressed or within the bottom *T*% of the less expressed, the subject also received a positive value of the signature. One hundred simulations were performed for each value of *T* and *G* used.

## CONFLICT OF INTERESTS

The author declares that he has no competing interests.

## AUTHORS' CONTRIBUTIONS

The author declares that the whole work was performed and written by his own.

## DATA AVAILABILITY STATEMENT

The statistics obtained and the results of simulations are included as supplementary files.

## ETHICAL COMPLIANCE

Because public data from third‐party organizations were used (https://portal.gdc.cancer.gov/), no explicit consent from participants is needed.

## CONSENT FOR PUBLICATION

By submission, the sole author waived the consent for publication.
