# Supplementary figures and images for: Integrative genomic analysis identifies associations of molecular alterations to APOBEC and BRCA1/2 mutational signatures in breast cancer
Source: Mol Genet Genomic Med. 2019 Jul 11;7(8):e810. doi: 10.1002/mgg3.810 (PMC6687632; doi:10.1002/mgg3.810)

## Mutation & CNA Data

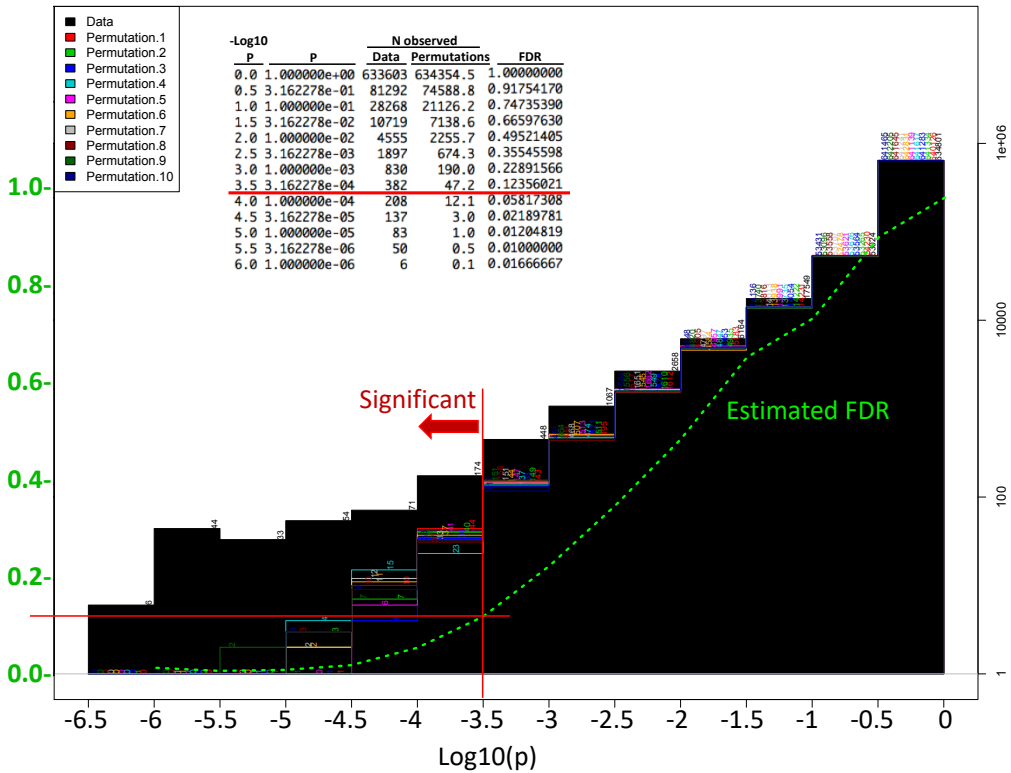

## Expression (mRNA, miRNA, RPPA)

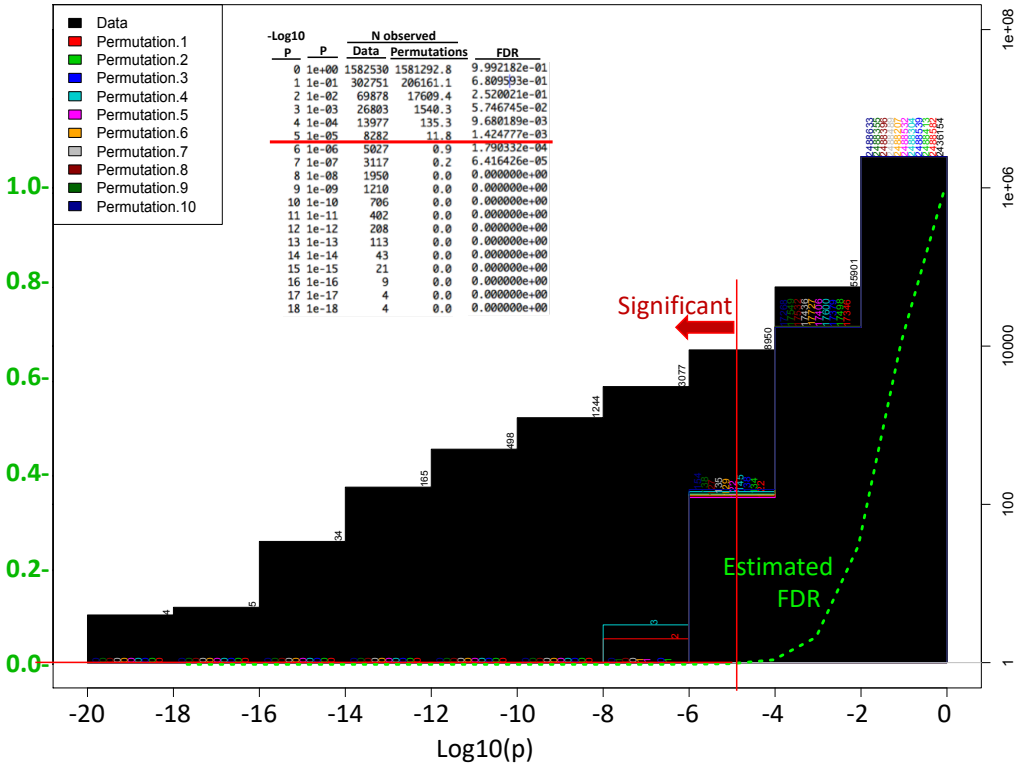

Supplement: Supplementary file 1 [file MGG3-7-e810-s001.pdf]

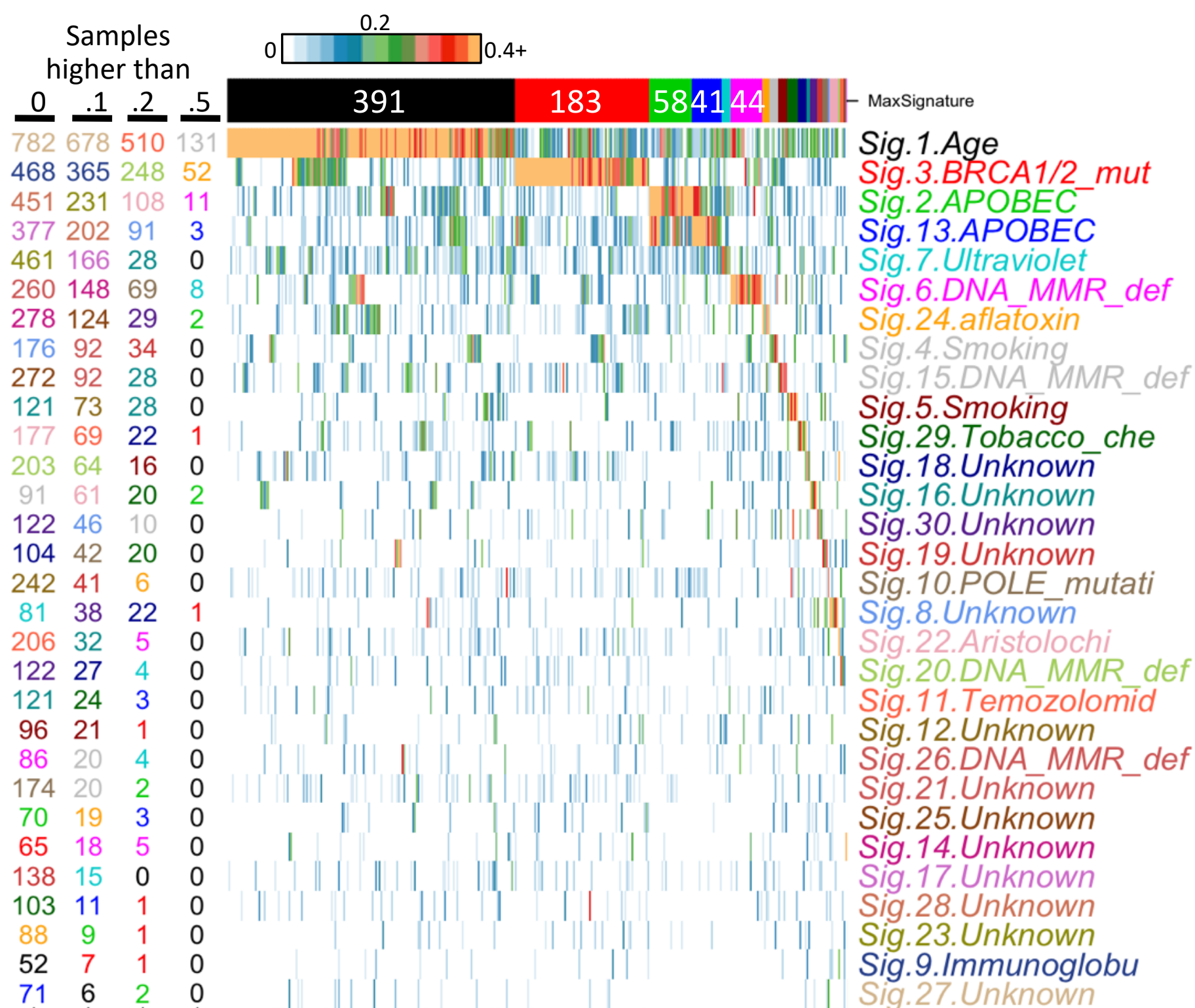

← Samples →

Supplement: Supplementary file 2 [file MGG3-7-e810-s002.pdf]

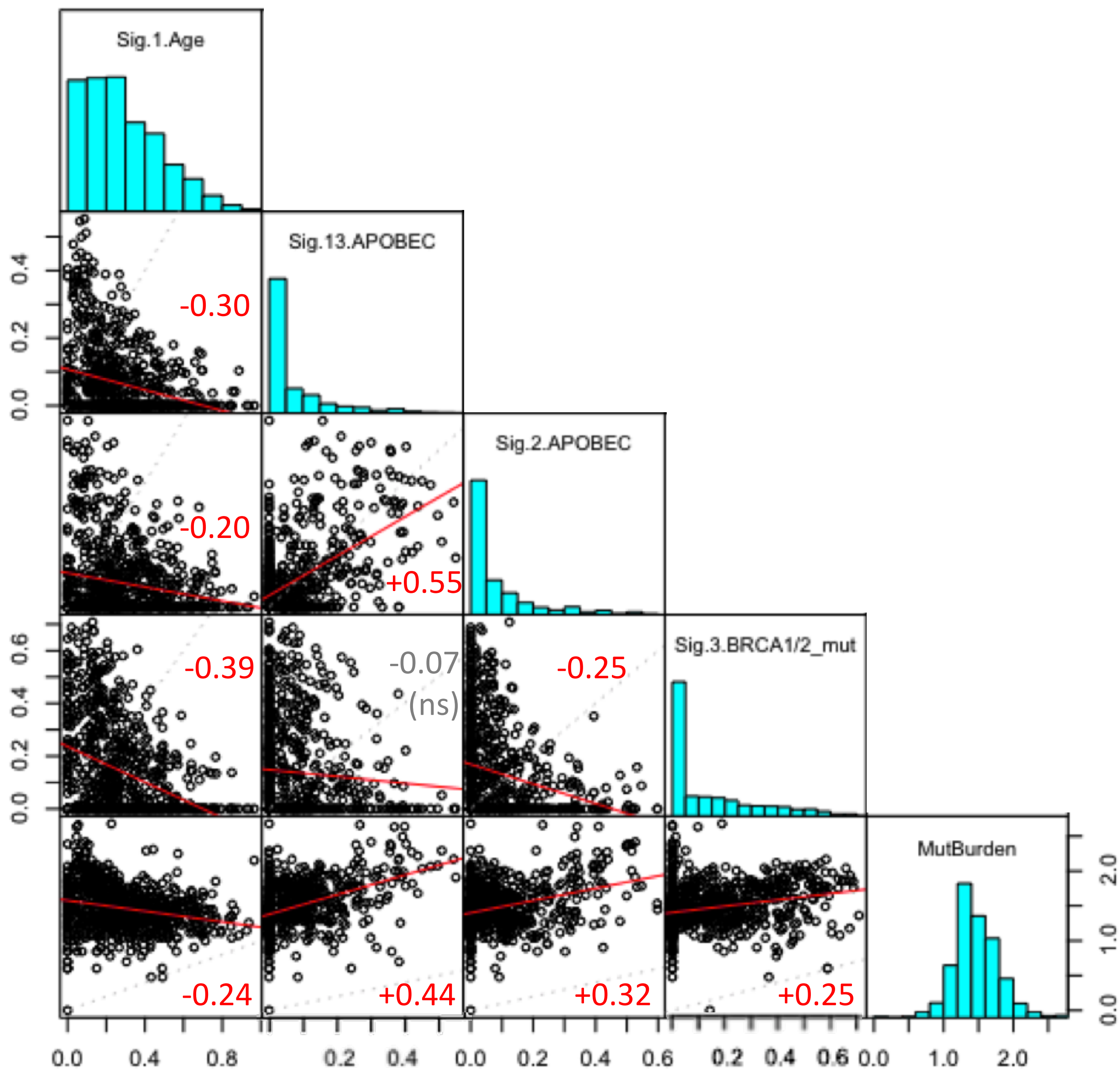

Supplement: Supplementary file 3 [file MGG3-7-e810-s003.pdf]

**(A)**

Sig.3. BRCA1/2 : Chromosome 4 Position (Mbp)

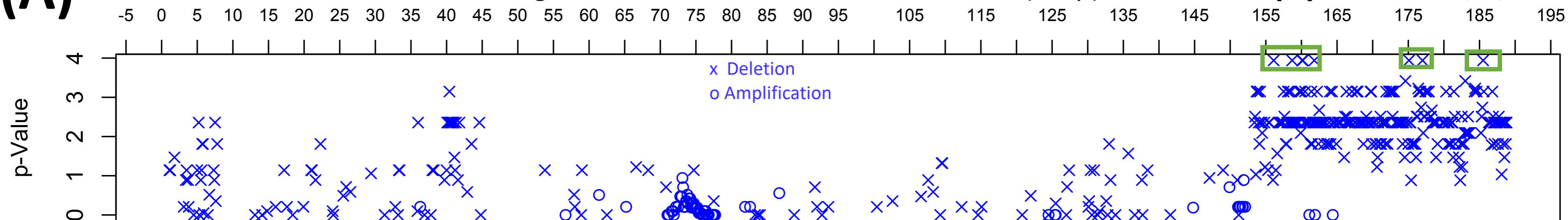**(B)**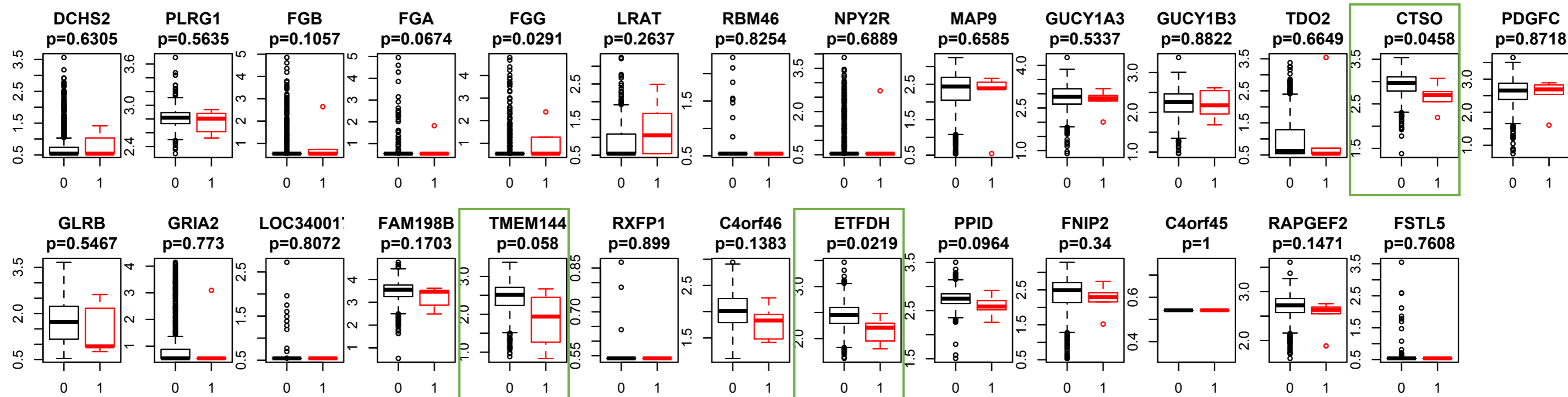**(C)**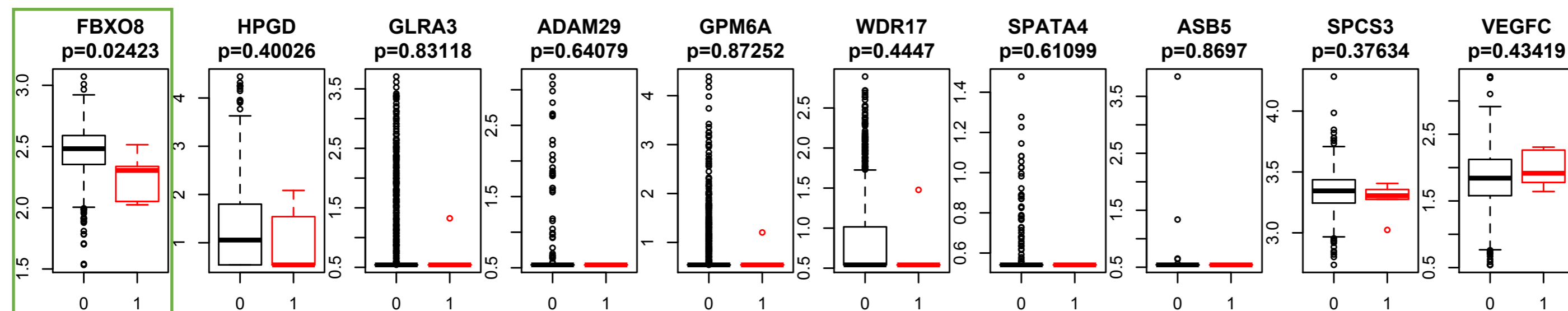**(D)**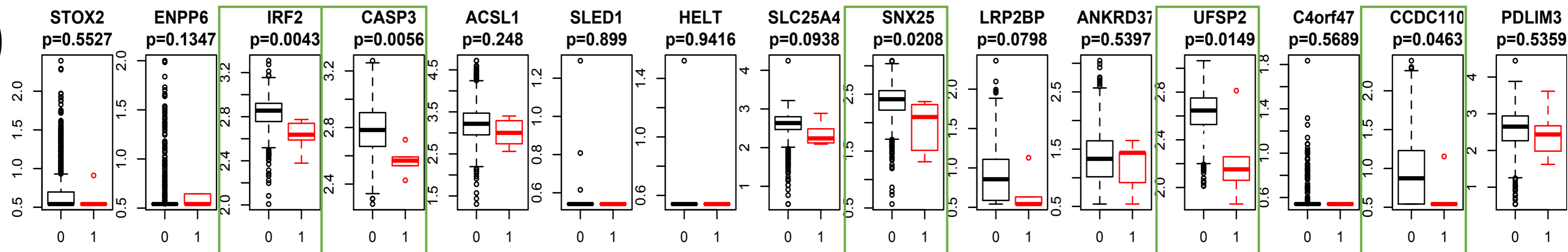

Supplement: Supplementary file 4 [file MGG3-7-e810-s004.pdf]

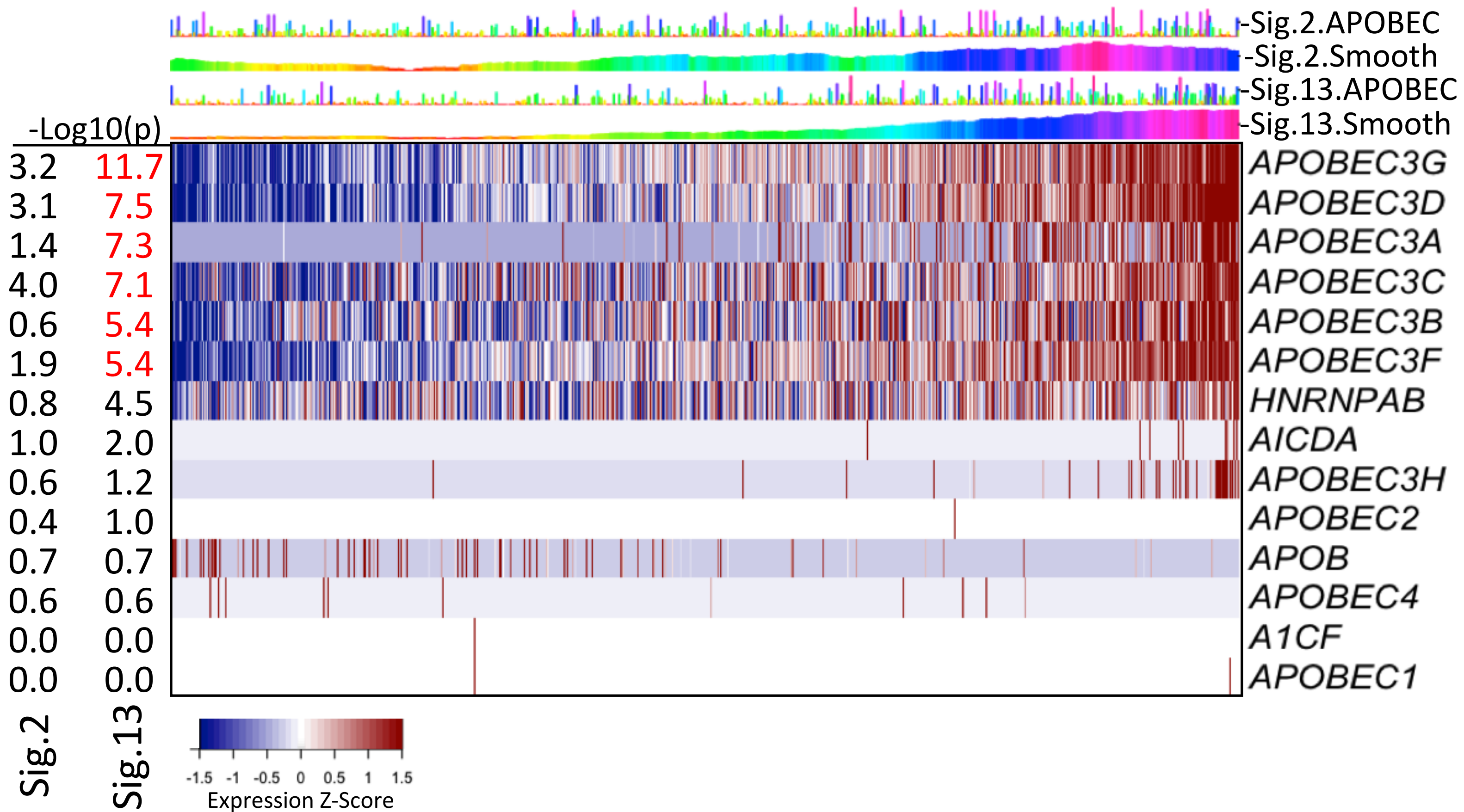

Supplement: Supplementary file 5 [file MGG3-7-e810-s005.pdf]

Co-Occurrence Sig.2.APOBEC

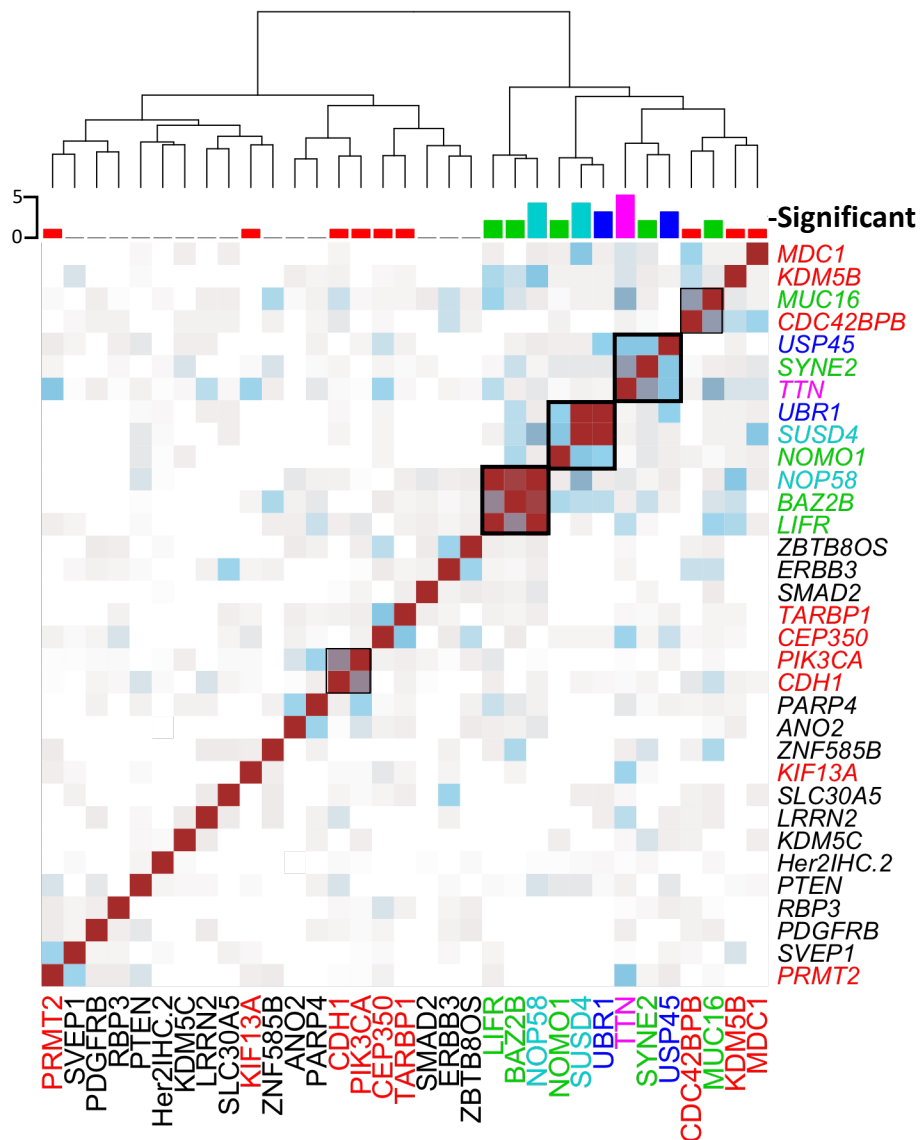

Co-Occurrence Sig.13.APOBEC

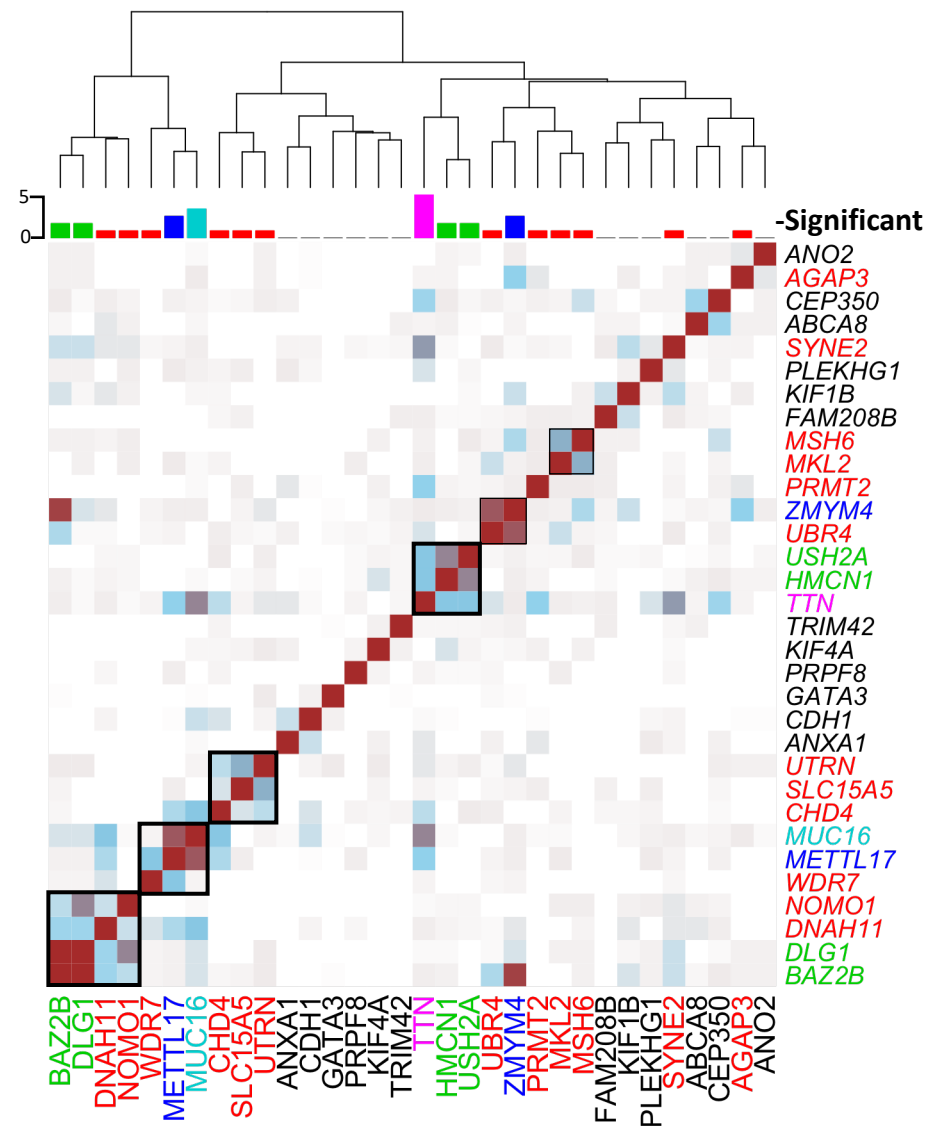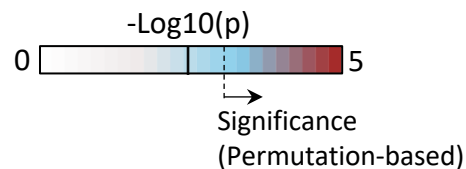

Supplement: Supplementary file 6 [file MGG3-7-e810-s006.pdf]

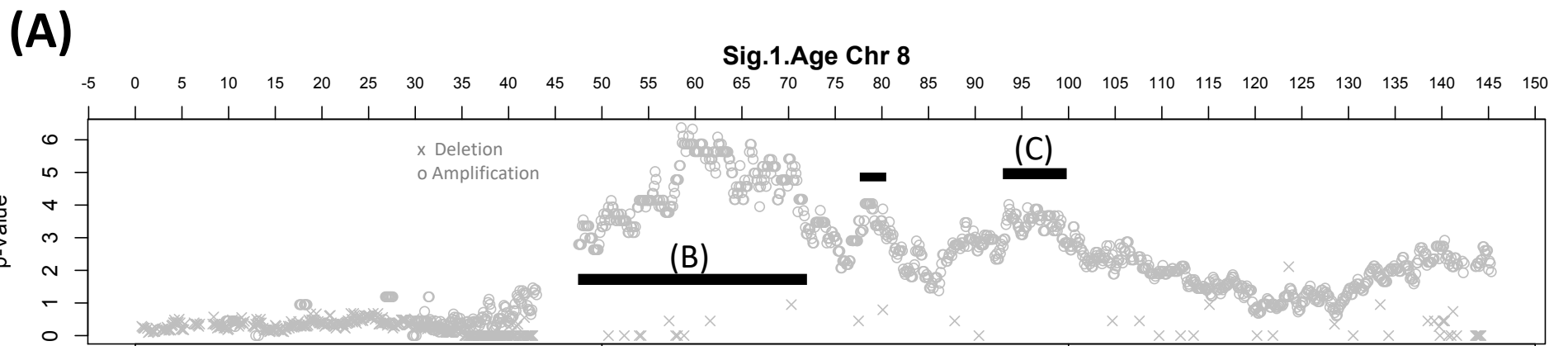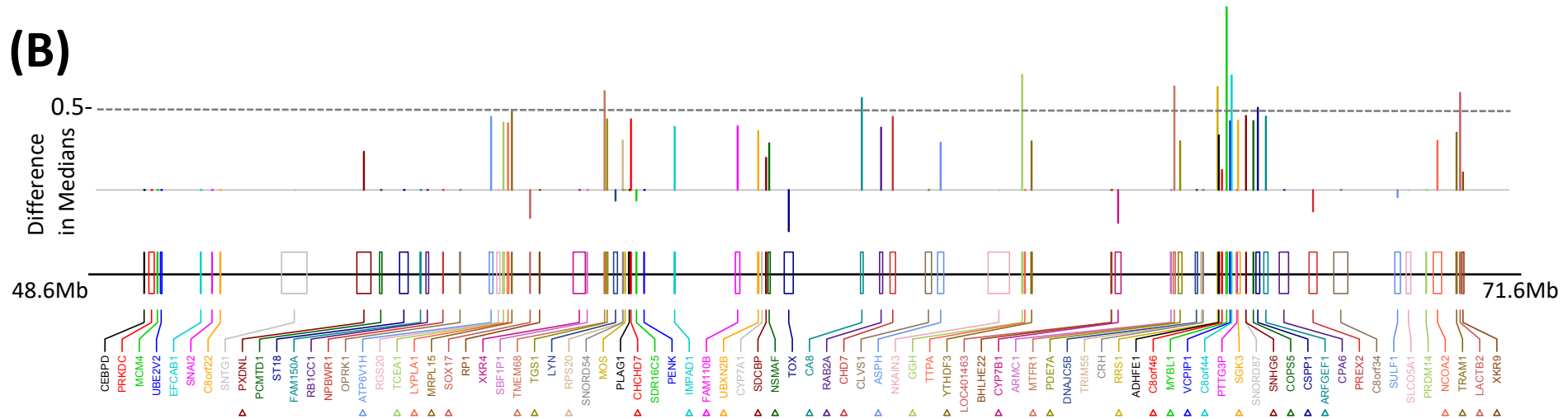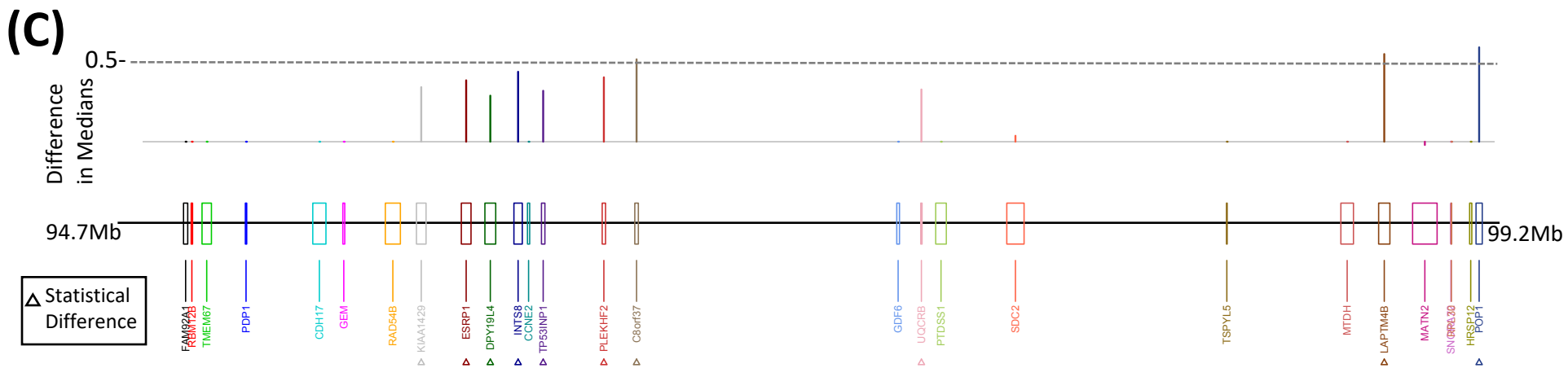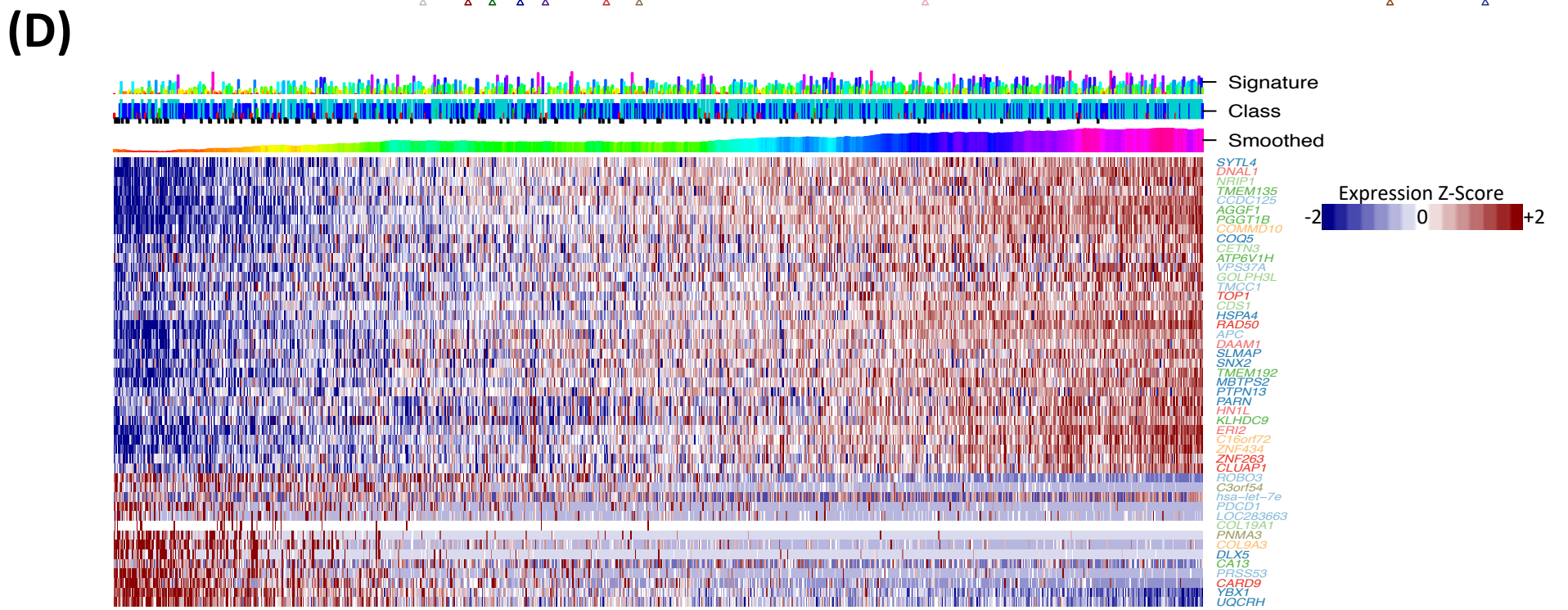

Supplement: Supplementary file 7 [file MGG3-7-e810-s007.pdf]
