# Supplementary material for: Integrative genomic analysis identifies associations of molecular alterations to APOBEC and BRCA1/2 mutational signatures in breast cancer
Source: Mol Genet Genomic Med. 2019 Jul 11;7(8):e810. doi: 10.1002/mgg3.810 (PMC6687632; doi:10.1002/mgg3.810)
Supplement: Supplementary file 8 [file MGG3-7-e810-s008.docx]

***Integrative genomic analysis identifies associations of molecular alterations to APOBEC and BRCA1/2 mutational signatures in breast cancer***

Victor Trevino

**Supplementary Table 1.** Performance of simulations.

| Parameters | n Subjects  Mean (SD) | Overall %  Sensibility | % Sensibility Expression | % Sensibility  CNA | % Sensibility  Mutations |
| --- | --- | --- | --- | --- | --- |
| T=5%, G=3 | 259 (32) | 32 | 49 | 16 | 13 |
| T=3%, G=3 | 174 (20) | 25 | 34 | 18 | 16 |
| T=1%, G=3 | 82 (10) | 52 | 6 | 98 | 98 |
|  |  |  |  |  |  |
| T=5%, G=5 | 417 (46) | 39 | 43 | 27 | 60 |
| T=3%, G=5 | 284 (38) | 33 | 29 | 24 | 48 |
| T=1%, G=5 | 142 (32) | 12 | 5 | 28 | 14 |
|  |  |  |  |  |  |
| T=5%, G=10 | 730 (53) | 44 | 29 | 55 | 64 |
| T=3%, G=10 | 520 (43) | 33 | 21 | 46 | 44 |
| T=1%, G=10 | 260 (16) | 16 | 5 | 27 | 26 |
